# Structural Basis of Host Autophagy-related Protein 8 (ATG8) Binding by the Irish Potato Famine Pathogen Effector Protein PexRD54*[Fn FN3]^♦^

**DOI:** 10.1074/jbc.M116.744995

**Published:** 2016-07-25

**Authors:** Abbas Maqbool, Richard K. Hughes, Yasin F. Dagdas, Nicholas Tregidgo, Erin Zess, Khaoula Belhaj, Adam Round, Tolga O. Bozkurt, Sophien Kamoun, Mark J. Banfield

**Affiliations:** From the ‡ Department of Biological Chemistry, John Innes Centre, and; the § Sainsbury Laboratory, Norwich Research Park, Norwich NR4 7UH, United Kingdom,; the ** Department of Life Sciences, Imperial College, London SW7 2AZ, United Kingdom,; ¶ EMBL Grenoble, 71 Avenue des Martyrs, CS 90181, 38042 Grenoble Cedex 9, France, and; ‖ EPSAM, Keele University, Keele ST5 5GB, United Kingdom

**Keywords:** autophagy, host-pathogen interaction, plant molecular biology, protein structure, protein-protein interaction, effector protein, plant pathogen

## Abstract

Filamentous plant pathogens deliver effector proteins to host cells to promote infection. The *Phytophthora infestans* R*X*LR-type effector PexRD54 binds potato ATG8 via its ATG8 family-interacting motif (AIM) and perturbs host-selective autophagy. However, the structural basis of this interaction remains unknown. Here, we define the crystal structure of PexRD54, which includes a modular architecture, including five tandem repeat domains, with the AIM sequence presented at the disordered C terminus. To determine the interface between PexRD54 and ATG8, we solved the crystal structure of potato ATG8CL in complex with a peptide comprising the effector's AIM sequence, and we established a model of the full-length PexRD54-ATG8CL complex using small angle x-ray scattering. Structure-informed deletion of the PexRD54 tandem domains reveals retention of ATG8CL binding *in vitro* and *in planta*. This study offers new insights into structure/function relationships of oomycete R*X*LR effectors and how these proteins engage with host cell targets to promote disease.

## Introduction

During selective autophagy, specific cellular constituents can be targeted to autophagic pathways for subcellular trafficking or degradation (1–3). The autophagy toolkit includes around 40 ATG (autophagy-related) proteins. Together, they help initiate, regulate, and form the constituents of autophagic pathways. The role of selective autophagy in the response to pathogen challenge in animal cells is increasingly being appreciated and includes direct elimination of microorganisms and control of immunity-related signaling (4, 5). In turn, microorganisms have developed mechanisms to perturb host-selective autophagy to either shut it down and promote infection (4, 5) or activate it and re-direct nutrients to the parasite (6). There is also evidence that membrane formation and trafficking, as controlled by ATG proteins, are exploited by numerous viruses (7). To date, the role of host-selective autophagy in host-microbe interactions has mostly been studied in mammals. The role of host-selective autophagy in plant-microbe interactions, and how it is manipulated by plant pathogens, remains poorly understood.

ATG8 is a ubiquitin-like protein that performs multiple functions in autophagy. It is cycled, via conjugation and deconjugation reactions, to the membrane lipid phosphatidylethanolamine, and this localization is important for autophagosome biogenesis (8). The intracellular animal pathogen *Legionella pneumophila* targets this process by delivering type IV secreted effector protein RavZ, which irreversibly deconjugates ATG8 from membranes and restricts autophagy (9). ATG8 also functions as an adaptor to interact with proteins containing an ATG8-interacting motif (AIM).^3^ AIM-containing proteins can serve as receptors for cargo destined for autophagosomes. The core AIM sequence is defined as Ω*XX*Ψ, where Ω is an aromatic amino acid (Trp, Tyr, or Phe); *X* is any residue, and Ψ is an aliphatic amino acid (Leu, Ile, and Val) (10–12). Frequently, residues just to the N terminus of the Ω*XX*Ψ motif are acidic in nature. Structural studies have elucidated how the AIM sequence binds ATG8, with key features including the Ω and Ψ residues binding within hydrophobic pockets, and the motif adopting a β-strand structure that extends the β-sheet of ATG8 (1, 13–15). It is generally thought that AIMs adopt a disordered or flexible conformation in the absence of a binding partner (11, 16). Mechanisms for pathogens to perturb host-selective autophagy include delivery of factors that interfere with recruitment of endogenous AIM-containing proteins to ATG8 or that re-direct additional cellular components to autophagosomes.

Filamentous plant pathogens cause devastating diseases of crops that are of both historical significance (17) and relevant to global agriculture today (18). *Phytophthora infestans*, the Irish potato famine pathogen, facilitates disease on its hosts by delivering effector proteins that modulate host cell processes to the benefit of the parasite (19), a strategy used by many biotrophic plant pathogens (20–22). Many putative *P. infestans* effectors contain a conserved N-terminal R*X*LR (Arg-Xaa-Leu-Arg) motif for host translocation (23). Furthermore, about half of these effectors are predicted to adopt the conserved WY domain fold in their C-terminal regions, which encodes their biochemical activity (24–26). Although recent studies have begun to elucidate the virulence-associated targets and functions of *P. infestans* R*X*LR effectors (27–34), these have yet to be identified for the vast majority of these proteins.

Recently, a *P. infestans* R*X*LR effector, PexRD54, which contains an AIM sequence Trp-Glu-Ile-Val “WEIV” positioned at the C terminus (residues 378–381), was identified (35). It was shown that PexRD54 specifically interacts with a member of the ATG8 family of proteins from potato, ATG8CL, *in vitro* and *in planta*. In plant cells, PexRD54 activates selective autophagy by increasing the number of ATG8CL-containing autophagosomes and stabilizing ATG8CL. Furthermore, PexRD54 was shown to antagonize the function of the host autophagy cargo receptor Joka2 by competing for binding with ATG8CL. As Joka2 contributed toward immunity against *P. infestans*, which was counteracted by PexRD54, it was concluded that this effector acts as an inhibitor of Joka2 function.

To better understand how PexRD54 interacts with potato ATG8CL to perturb host-selective autophagy, we have investigated the structural basis of effector-host target interaction. We determined the crystal structures of PexRD54 and ATG8CL in complex with the C-terminal AIM peptide of this effector. We also obtained a structure of the PexRD54-ATG8CL complex by docking the crystal structures into an envelope derived from solution scattering data. Site-directed mutagenesis of the PexRD54 C-terminal AIM region, and ATG8CL binding to a PexRD54 AIM-based peptide array, mapped the key residues that define the PexRD54-ATG8CL interface. Finally, we used structure-informed deletions to show that the WY domains of PexRD54 are dispensable for ATG8CL binding suggesting an alternative function for these domains. Together, these data provide a mechanistic understanding of how translocated effectors engage with their host targets and offer new methods for engineering control of plant diseases.

## Results

### 

#### 

##### PexRD54 Forms a Stable Complex with ATG8CL in Vitro

**FIGURE 1.**
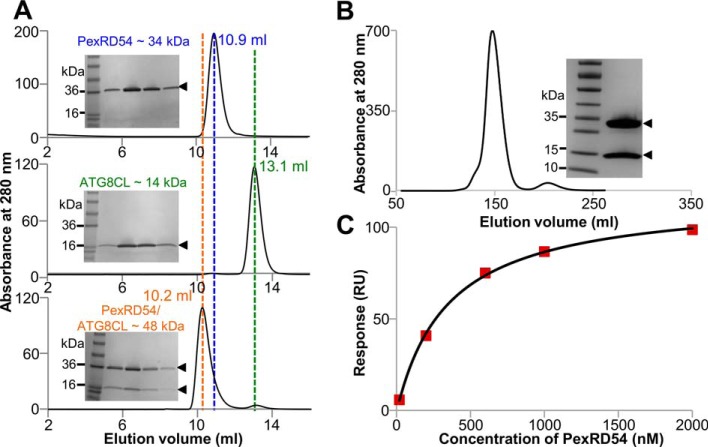
**Interaction of PexRD54 and ATG8CL proteins *in vitro*.** *A,* analytical gel filtration traces obtained for PexRD54 (*top*), ATG8CL (*middle*), and a 1:1 mixture of the complex (*bottom*). *Insets* show SDS-polyacrylamide gels of the fractions collected across the elution peaks. *B,* gel filtration trace derived from preparative purification of the PexRD54-ATG8CL complex following co-expression in *E. coli. Inset,* SDS-polyacrylamide gel containing purified complex. *C,* binding curve derived from SPR single cycle kinetics data for PexRD54 binding to ATG8CL.

To investigate complex formation between PexRD54 and ATG8CL, we expressed both proteins separately in *Escherichia coli* and purified them to homogeneity (Fig. 1*A*). To determine whether the two proteins form a stable complex in solution, we mixed them in an equimolar ratio prior to injection on a Superdex S75 10/300 analytical gel filtration column and compared the resulting elution volume to the elution volumes of the individual proteins. As shown in Fig. 1*A*, PexRD54 elutes at 10.9 ml and ATG8CL at 13.1 ml when these proteins are run independently. After mixing, a new peak at an earlier elution volume (10.2 ml) is apparent, and SDS-PAGE analysis shows this peak contains both proteins. This shift in the elution peak is indicative of complex formation and that this complex is stable over the time course of the experiment. Based on a calibration curve, elution volumes from this column of 10.9, 13.1, and 10.2 ml correspond to ∼44, ∼18, and ∼58 kDa. All these represent overestimates of the predicted molecular masses of the proteins on their own or in complex (PexRD54 ∼34 kDa, ATG8CL ∼15 kDa, and PexRD54-ATG8CL complex ∼49 kDa) but indicate monomeric forms of each state exist in solution.

Next, we determined whether the PexRD54-ATG8CL complex could be formed on purification following co-expression in *E. coli*. We cloned PexRD54 and ATG8CL into different expression vectors, with only the ATG8CL containing a His_6_ tag (see under “Experimental Procedures”). Following expression and preparative tandem immobilized metal affinity chromatography/gel filtration chromatography of the clarified cell lysate, a single peak was obtained at an elution volume consistent with a complex between PexRD54 and ATG8CL (Fig. 1*B*, an elution volume of 151 ml on this column corresponds to ∼50 kDa, predicted molecular mass of the complex is ∼49 kDa). SDS-PAGE analysis of the fractions confirmed the presence of both proteins (Fig. 1*B*). This shows that a complex between PexRD54 and ATG8CL is likely formed in cells and can be purified from cell culture directly.

**FIGURE 2.**
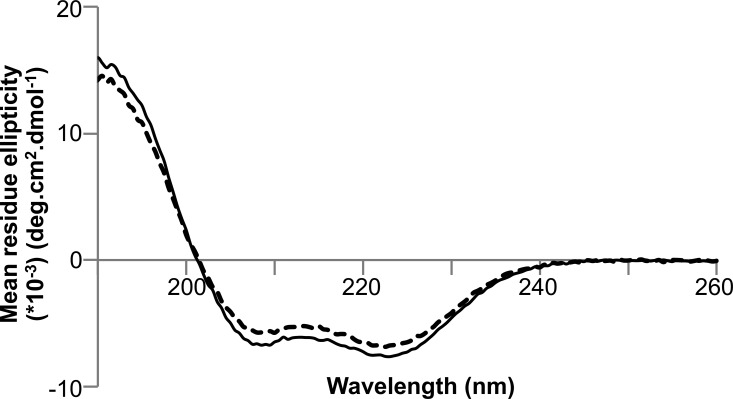
**CD spectra of PexRD54.** Far-UV CD spectra of wild-type PexRD54 (*solid line*) and its variant PexRD54^378-AEIA-381^ (*dashed line*) confirming similar secondary structure content (predominantly α-helical).

Finally, we used surface plasmon resonance (SPR) to investigate the affinities of complex formation between PexRD54 and ATG8CL (Fig. 1*C*). Using this technique, we determined that PexRD54 binds to ATG8CL with a *K_d_* of 388 ± 47 nm. The AIM motif disrupting PexRD54^378-AEIA-381^ variant (where the Trp and Val of the “WEIV” AIM motif are replaced by alanine) did not bind to ATG8CL using SPR, consistent with previous results (35). The overall fold of the PexRD54^378-AEIA-381^ variant was equivalent to wild-type protein as assessed by circular dichroism (CD) spectroscopy (Fig. 2).

##### PexRD54 Is a Tandem Repeat WY Domain Effector with a Disordered C-terminal AIM

**FIGURE 3.**
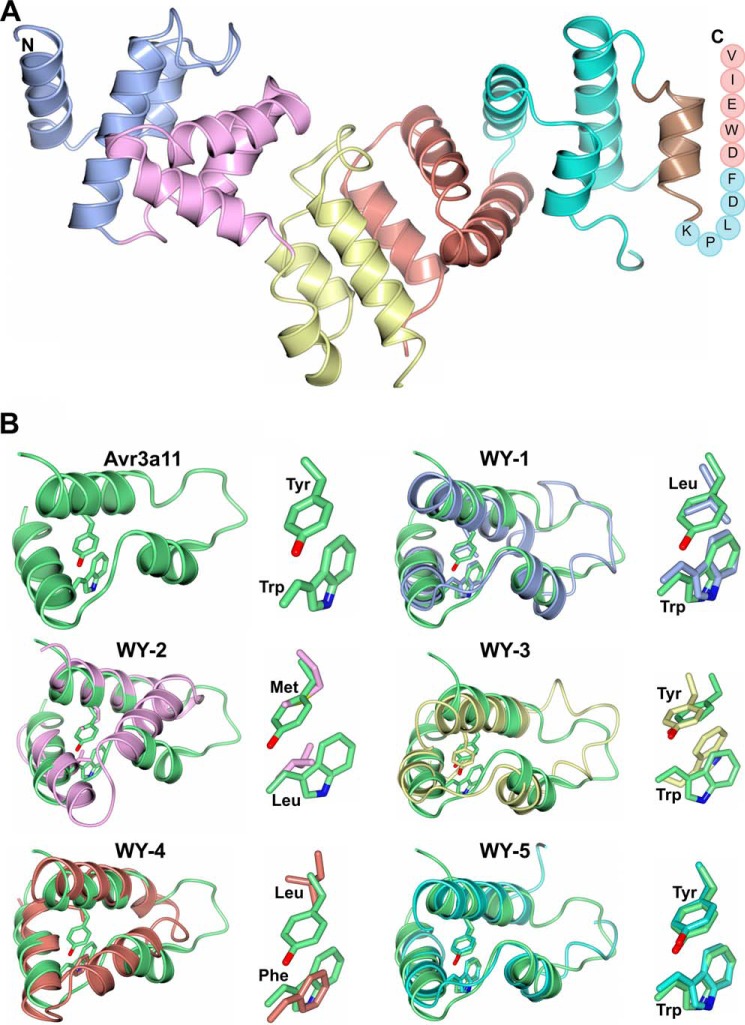
**Crystal structure of PexRD54.** *A,* schematic representation of the crystal structure of PexRD54 showing the five tandem WY domains (*blue, magenta, yellow, coral,* and *cyan*) and the disordered AIM motif at the C terminus (*circles* with *single letter* amino acid codes shown). The N and C termini are labeled. *B,* superimposition of the WY domains of AVR3a11 (*top left, green*) on the WY domains from PexRD54. The characteristic hydrophobic residues of each WY domain are also shown in *stick* representation. The PexRD54 WY domains are colored as in *A*.

To discover the molecular architecture of PexRD54, we determined the crystal structure of the effector domain of this protein (residues Val-92 to Val-381) at 2.90 Å resolution. Although PexRD54 could be crystallized alone, the crystal that gave rise to the best x-ray dataset was obtained from a sample including both PexRD54 and ATG8CL after co-expression in *E. coli* (see under “Experimental Procedures”). Although SDS-polyacrylamide gel analysis of dissolved crystals showed that both proteins were present in these crystals, no electron density for ATG8CL was observed. The structure of PexRD54 was solved using single wavelength anomalous diffraction, and the final model was refined to final *R*_work_ and *R*_free_ values of 23.1 and 25.6%, respectively (Table 1). Inspection of the packing of PexRD54 revealed that ATG8CL could be accommodated in the crystal, within a region of unaccounted for space near the C terminus of the effector. The structure of PexRD54 includes 16 α-helices (Fig. 3*A* and supplemental video 1). Five N-terminal residues (92–96), the residues in two loops (248–250 and 331–334), and 11 C-terminal residues (371–381), which include the AIM motif, were not included in the final model due to poor electron density in these regions.

**TABLE 1:** **PexRD54/ATG8CL x-ray data collection and refinement statistics**

	PexRD54		ATG8CL native
	Native	Iodide	
**Data collection statistics**			
Wavelength (Å)	0.9795	2.0	0.9795
Space group	*P*3_1_21	*P*3_1_21	*I*4_1_32
Cell dimensions			
*a*, *b*, *c* (Å)	89.16, 89.16, 144.32	91.67, 91.67, 144.66	172.80, 172.80, 172.80
Resolution (Å)*^a^*	77.21–2.90 (2.90–2.98)	79.39–3.50 (3.50–3.59)	86.09–1.90 (1.90–1.95)
*R*_merge_ (%)	7.0 (134.9)	13.9 (116.8)	13.0 (132.5)
*I*/σ*I*	24.9 (2.9)	22.5 (3.9)	27.4 (3.4)
Completeness (%)			
Overall	99.8 (99.7)	99.9 (100)	100 (100)
Anomalous		99.9 (99.8)	
Unique reflections	15,256 (1132)	9319 (676)	34,386 (2623)
Redundancy			
Overall	12.1 (12.3)	31.6 (29.2)	32.8 (31.9)
Anomalous		16.8 (15.1)	
*CC*(1/2) (%)*^a^*	99.9 (79.6)	99.9 (91.3)	100 (86.4)

**Refinement and model statistics**			
Resolution (Å)	77.21–2.90 (2.98–2.90)		86.09–1.90 (1.95–1.90)
*R*_work/_*R*_free_ (%)	23.1/25.6 (40.5/32.5)		17.6/19.9 (24.2/25.3)
No. of atoms			
Protein	2224		235
*B*-Factors			
Protein	98.9		24.00
Root mean square deviations			
Bond lengths (Å)	0.007		0.011
Bond angles (°)	1.047		1.50
Ramachandran plot (%)*^b^*			
Favored	94.25		98.71
Allowed	5.75		1.29
Outliers	0		0
MolProbity Score	1.45 (100th percentile)		1.14 (100th percentile)

*^a^* The highest resolution shell is shown in parentheses.

*^b^* Data are as calculated by MolProbity.

Previous bioinformatics analysis predicted the presence of multiple WY domains in PexRD54 (24). Our structural analysis revealed that PexRD54 includes five tandem WY domains that pack to form an elongated molecule (Fig. 3*A*). This is a conformation not yet observed for R*X*LR effectors with multiple WY domains. The WY domain is a conserved structural unit consisting of three α-helices and two characteristic hydrophobic amino acids, frequently W (Trp) and Y (Tyr), which contribute to a stable hydrophobic core (24, 25). Structural superposition of the archetypal WY domain of the *Phytophthora capsici* R*X*LR-WY effector AVR3a11 on each of the WY domains of PexRD54 is shown in Fig. 3*B*, with root mean square deviations derived from each superposition given in Table 2. As more structures are determined, it is increasingly clear that WY domains can tolerate variations at the Trp and Tyr positions, while maintaining the hydrophobic core and overall fold. This is in addition to the remarkable overall structural conservation among WY domains despite a lack of pairwise sequence identity, which is as low as 13% between PexRD54 and AVR3a11 (Table 2).

**TABLE 2:** **Root mean square deviations (r.m.s.d.) derived from the overlays shown in Fig. 3*B*, including the number of carbon atoms in the overlay, the identity of the “WY” amino acids, and percentage sequence identity to AVR3a11**

	r.m.s.d.	Residue range	WY amino acids	Sequence identity to AVR3a11
	Å			%
WY-1	1.81/37	Ser-97–Gly-150	WL	13
WY-2	2.35/32	Asn-151–Gly-198	LM	18
WY-3	2.89/39	Asn-199–Asn-247	WY	16
WY-4	2.80/41	Phe-251–Ser-299	FL	14
WY-5	1.73/41	Ser-302–Ile-354	WY	20

##### Host Protein ATG8CL Binds the PexRD54 AIM Sequence via Two Hydrophobic Pockets

In the PexRD54 structure, we did not observe the last 10 amino acids that contain the AIM motif, or the ATG8CL protein itself, in the electron density. Therefore, to visualize the interaction between PexRD54 and ATG8CL, we determined the crystal structure of ATG8CL in complex with a PexRD54 C-terminal pentapeptide. This pentapeptide includes the AIM motif, with residues Asp-377–Trp-378–Glu-379–Ile-380–Val-381. To produce crystals of ATG8CL + pentapeptide, we used an ATG8CL construct lacking four N-terminal residues and five C-terminal residues.

The structure of the complex was solved by molecular replacement and refined to 1.90 Å with final *R*_work_ and *R*_free_ values of 17.6 and 19.9%, respectively (Table 1). Positive difference electron density within the likely AIM binding region of ATG8CL indicated the presence of bound pentapeptide. The final model contains two molecules of ATG8CL + pentapeptide in the asymmetric unit. The electron density maps for both complexes were of equivalent quality, and subsequent analysis focuses on one representative monomer.

**FIGURE 4.**
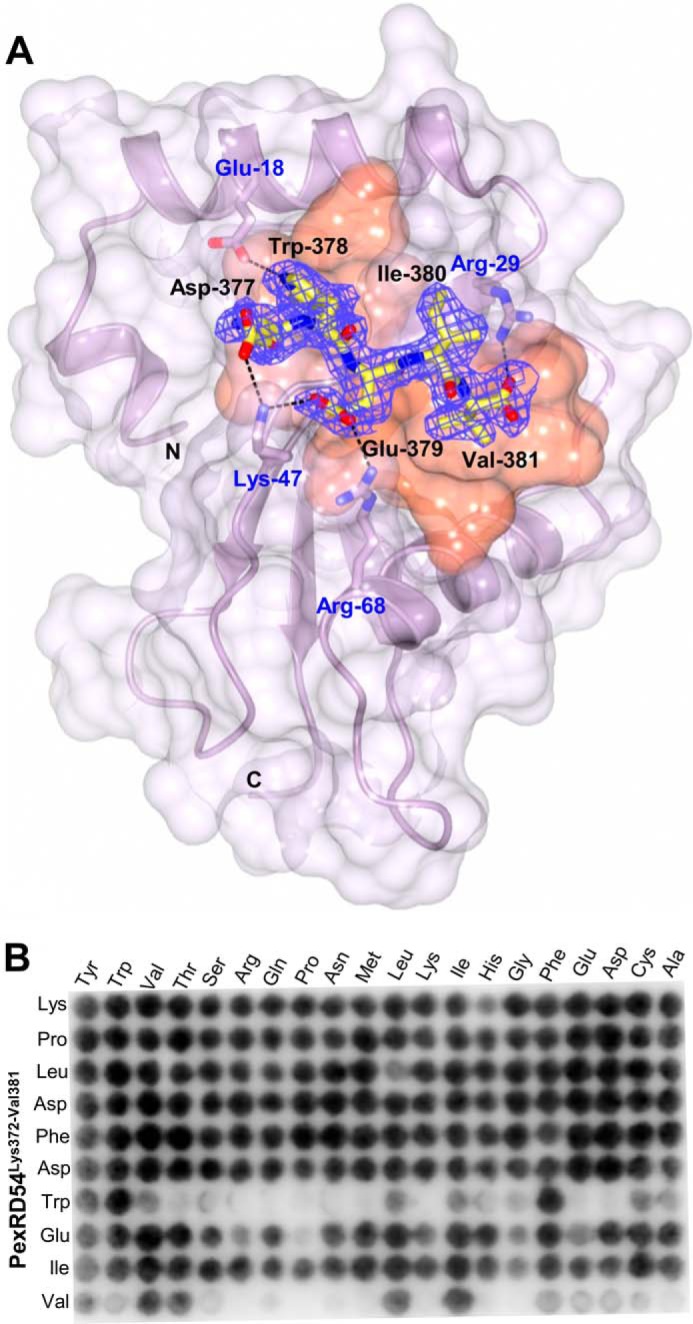
**Crystal structure of ATG8CL bound to the PexRD54(377–381)-peptide and specificity of peptide binding.** *A,* schematic representation of ATG8CL/PexRD54(377–381)-peptide complex highlighting key interactions. ATG8CL is shown in *magenta* schematic representation with the molecular surface that contacts the PexRD54(377–381)-peptide shown in *orange*. The PexRD54(377–381)-peptide is shown as *sticks* with *yellow* carbon atoms. The electron density omit map of the peptide ligand (*F*_obs_ − *F*_calc_ map) is shown in *blue mesh* and contoured at 2 σ. Electrostatic interactions are indicated with *black dashed lines. B,* results of the peptide array analyzing the effect of single amino acid substitutions (*top*) at all positions of 10-mer peptide of PexRD54 (Lys-372–Val-381, *side*). GST-tagged ATG8CL was visualized using an anti-GST-HRP antibody.

The structure of ATG8CL contains two domains, an N-terminal helical domain (α1 and α2) and a C-terminal domain that adopts a β-grasp (ubiquitin-like) fold of four β-strands (β1–β4) flanked by two helices (α3 and α4) (Fig. 4*A*). ATG8CL adopts a very similar structure to that observed for ATG8s from other organisms. For example, ATG8CL overlays on the structures of GATE-16 (Protein Data Bank code 1EO6, 60% sequence identity with ATG8CL) and GABARAP (Protein Data Bank code 4XC2, 57% sequence identity with ATG8CL) with a root mean square deviation of 0.8 and 0.9 Å, respectively, for 115 α-carbons.

In the complex, the pentapeptide adopts an extended conformation forming a parallel β-sheet with β2 of ATG8CL. The peptide binds within a narrow channel at the surface of ATG8CL via hydrophobic and hydrogen bond interactions (Fig. 4*A*). The side chain of PexRD54 Trp-378 is contained within a hydrophobic pocket formed at the interface between the β-grasp and N-terminal helical domains of ATG8CL, whereas the side chain of PexRD54 Val-381 binds a distinct hydrophobic pocket between β2 and an adjacent helix on the C-terminal domain of ATG8CL (Fig. 4*A*). In addition to hydrophobic interactions, the indole nitrogen of Trp-378 forms a hydrogen bond with the side chain of ATG8CL Glu-18 (Fig. 4*A*). The side chain of PexRD54 Glu-379 makes hydrogen bonds and ionic interactions with the side chains of ATG8CL Lys-47 and ATG8CL Arg-68 (Fig. 4*A*). Another prominent ionic interaction is formed between the side chain of PexRD54 Asp-377 and ATG8CL Lys-47 (Fig. 4*A*).

##### Molecular Envelope of the Full-length PexRD54 and ATG8CL Complex

Despite having determined the crystal structures of PexRD54 and of ATGCL bound to the PexRD54 AIM motif pentapeptide, structural information on how the full-length proteins interact was still lacking. To gain insight into this, we collected solution x-ray scattering data (small angle x-ray scattering (SAXS)) of both PexRD54 alone and the PexRD54-ATG8CL complex following co-expression and purification as described previously.

**FIGURE 5.**
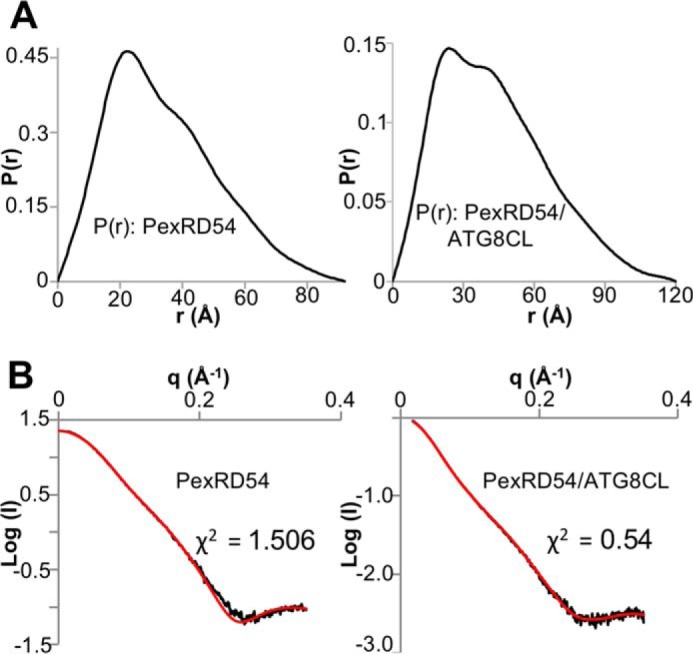
**Analysis of SAXS data.** *A, P*(*r*) distribution curves used for *ab initio* modeling. *Left,* PexRD54; *right,* PexRD54-ATG8CL complex. *D*_max_ was set at 92 nm (PexRD54) and 120 nm (PexRD54/ATG8CL complex). Data were cropped at 0.35 Å^−1^ for analysis. *B, left,* fit of the theoretical scattering curve of PexRD54 from CRYSOL (*red*) to the PexRD54 scattering data (*black*). *Right,* fit of the theoretical scattering curve of the PexRD54-ATG8CL complex from CORAL (*red*) to the PexRD54-ATG8CL scattering data (*black*).

Analysis of the solution scattering data (“Experimental Procedures”) revealed that the PexRD54 particle has a radius of gyration of 26.1 Å (from Guinier analysis) or 26.7 Å (from *P*(*r*) function (Fig. 5*A, left*)), with a maximal dimension (*D*_max_) of 92 Å. This compares well with the maximal dimension in the crystal structure of ∼87 Å. The predicted molecular mass from the Porod-Debye analysis is 26–34 kDa, which is close to the mass determined by LC-MS (34.023 kDa). The PexRD54-ATG8CL complex particle has a radius of gyration of 32.6 Å (from Guinier analysis) or 34.1 Å (from *P*(*r*) function (Fig. 5*A*, *right*)) with a D_max_ of 120 Å. The predicted molecular mass from the Porod-Debye analysis is 41–54 kDa, and the mass of the proteins in the complex as determined by LC-MS (48.694 kDa) fits well within this range. *Ab initio* shape reconstructions of the particles were generated, and the crystal structure of PexRD54 (for the PexRD54 data) was docked into its envelope (Figs. 5*B*, *left,* and 6, *A* and *B*). A complex between PexRD54 and ATG8CL + pentapeptide consistent with the scattering data was generated using CORAL (36) and subsequently docked into the appropriate envelope (Figs. 5*B*, *right,* and 6, *A* and *C*). The latter model provides a molecular snapshot of a *P. infestans* translocated effector protein bound to a host target.

**FIGURE 6.**
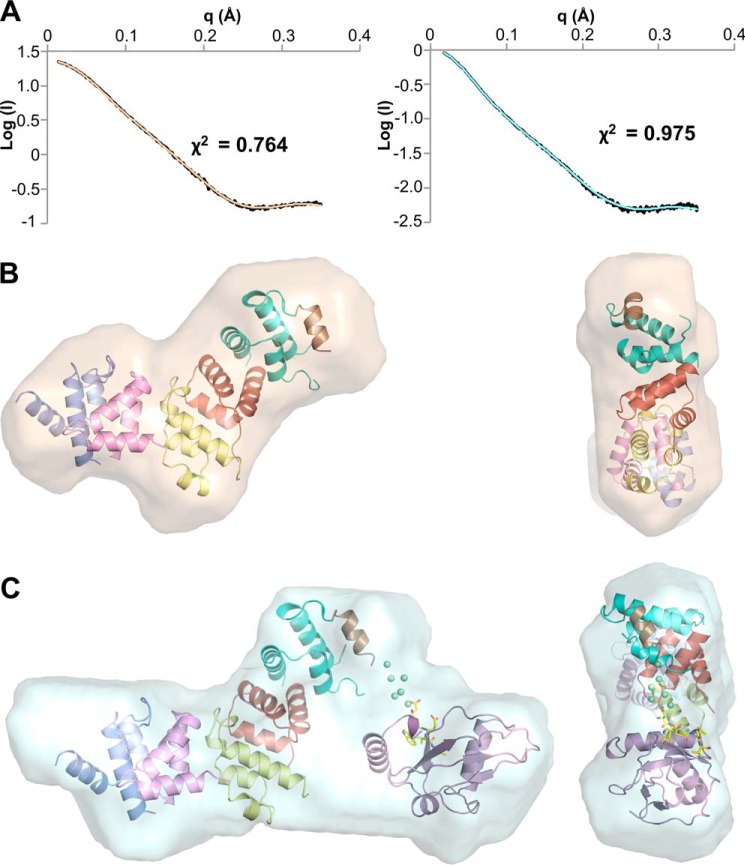
**PexRD54 and PexRD54-ATG8CL complex analyzed by small angle x-ray scattering.** *A,* fits of the most probable (lowest NSD) dummy atom models from DAMMIN for PexRD54 (*left*) and PexRD54/ATG8CL (*right*). The fit to the experimental data (in *black*) is shown in *wheat* and *cyan*, respectively, with χ^2^ shown as an *inset. B,* superposition of the crystal structure of PexRD54 with the most probable *ab initio* envelope of PexRD54 (*wheat surface*). *C,* superposition of the CORAL rigid body model of PexRD54/ATG8CL + pentapeptide with the most probable *ab initio* envelope of the complex (*cyan surface*). For *B* and *C,* two views are shown, face-on (*left*) and end-on (*right*). The fits shown in *A* and the envelopes shown in *B* and *C* are from the same run of DAMMIN.

##### Characterization of the PexRD54 AIM Region Binding to ATG8CL

To build on the structural studies above, we used two complementary biochemical approaches to investigate the role of individual residues in the AIM region of PexRD54 in binding to ATG8CL.

**FIGURE 7.**
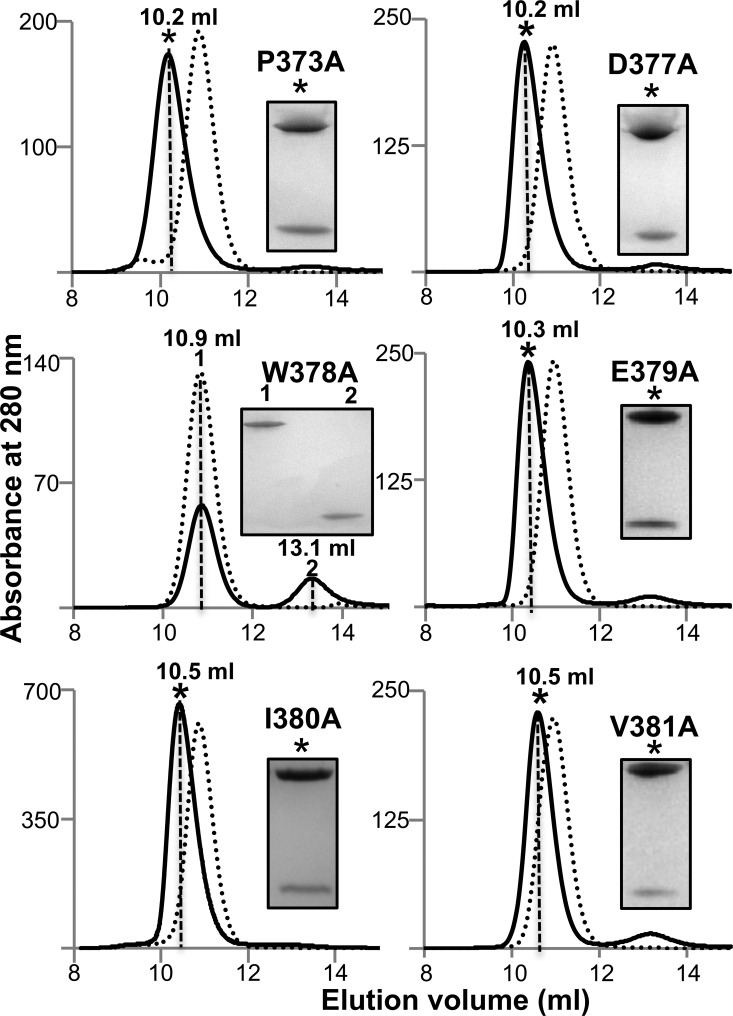
**Analysis of the interaction between PexRD54 variants and ATG8CL by gel filtration.** Analytical gel filtration traces were obtained for PexRD54 variants mutated in the AIM region and incubated with ATG8CL (1:1 mixture). *Insets* show SDS-polyacrylamide gels of the fractions at the elution peaks as marked by the *dashed lines*.

First, we used alanine-scanning mutagenesis to substitute Ala at six positions in the PexRD54 AIM region, Pro-373, Asp-377, Trp-378, Glu-379, Ile-380, and Val-381. Each of these proteins was expressed and purified as described for wild type. We then used analytical gel filtration to qualitatively assay whether these variants support complex formation with ATG8CL. As predicted, we did not observe interaction of PexRD54 W378A with ATG8CL (Fig. 7). For each of the other mutations, we still observed an interaction with ATG8CL, including PexRD54 V381A. Second, we designed a nitrocellulose-anchored peptide array of 200 variant AIM peptides, based on the final 10 amino acids of PexRD54, where each amino acid was changed to all other possible amino acids. The peptides were anchored at the N terminus to best mimic the presentation of the PexRD54 AIM region to ATG8CL. We visualized ATG8CL binding to the peptide array using an ATG8CL fusion with glutathione *S*-transferase (GST) and a His tag (see “Experimental Procedures”), followed by incubation with an anti-GST-HRP antibody (Amersham Biosciences) and detection of chemiluminescence (Fig. 4*B*). The results of the peptide array clearly highlight the importance of the hydrophobic residues 378 and 381 of the PexRD54 AIM motif (Trp and Val) in binding ATG8CL. For position 378, the strongest binding was seen for Trp and Phe, with limited binding of Tyr and the aliphatic amino acids. Position 381 favors the bulky aliphatic amino acids, with limited binding also observed by bulky hydrophobic residues. Interestingly, with the exception of Pro at position 379, any amino acid can be accommodated at positions 379 and 380, and binding is still observed. Furthermore, any amino acid can be accommodated at positions 372–377 without a significant reduction in binding, suggesting that these residues may only act as a linker between the WY domain region of PexRD54 and the C-terminal AIM motif.

##### WY Domains of PexRD54 Are Dispensable for the Interaction with ATG8CL in Vitro and in Planta

**FIGURE 8.**
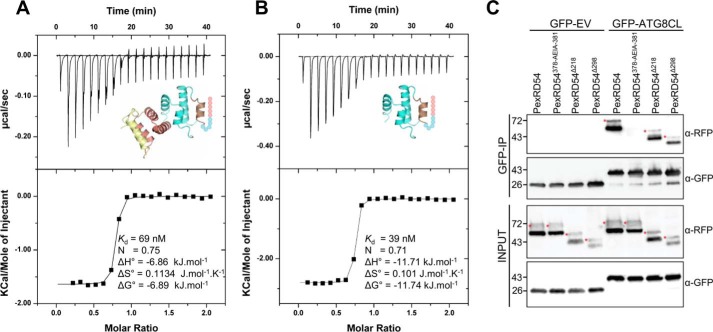
**Interaction of PexRD54^Δ218^ and PexRD54^Δ298^ with ATG8CL *in vitro* and *in planta*.** The binding affinities of PexRD54^Δ218^ (*A*) and PexRD54^Δ298^ (*B*) to ATG8CL were determined by ITC. Following a heats-of-dilution correction, a single-site binding model was used to fit the data using the MicroCal Origin software (data are shown on the *top*, with the fit on the *bottom*). The *insets* in the *top panel* depict the PexRD54 truncation used in the experiment, colored as in Fig. 3*A. C,* validation of PexRD54^Δ218^ and PexRD54^Δ298^ interaction with ATG8CL in plant cells by co-immunoprecipitation. *Red asterisks* indicate expected band sizes of the PexRD54 constructs. Degradation is due to autophagy, as seen previously (35).

**FIGURE 9.**
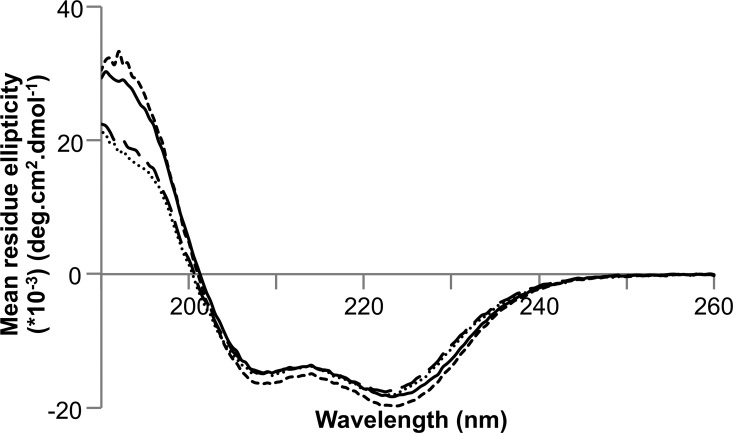
**CD spectra of truncated PexRD54 constructs.** Far-UV CD spectra of PexRD54^Δ218^ (*solid line*), PexRD54^Δ298^ (*long dash line*), PexRD54^Δ218AEIA^ (*short dashed line*), and PexRD54^Δ298AEIA^ (*dotted line*) variants confirming a similar secondary structure composition (predominantly α-helical).

Although the AIM region of PexRD54 appears necessary and sufficient for the interaction with ATG8CL, we explored whether the WY domains of PexRD54, which include ∼96% of the protein expressed here, impact the binding of the effector to ATG8CL. For this, we produced two structure-informed deletions of PexRD54, removing either the first three WY domains (but leaving the C-terminal helix of WY-3, which forms an N-terminal extension of WY-4), generating PexRD54^Δ218^, or the first four WY domains (leaving only WY-5), producing PexRD54^Δ298^ (Fig. 8, *A* and *B*). These proteins were expressed and purified as for wild-type PexRD54 and confirmed to be predominantly α-helical by CD spectroscopy (Fig. 9). We used ITC to calculate the affinity of interaction for these constructs with ATG8CL, which gave a *K_d_* of 69 nm for PexRD54^Δ218^ and a *K_d_* of 39 nm for PexRD54^Δ298^ (Fig. 8, *A* and *B*). These values are broadly in line with the *K_d_* of 383 nm obtained for the ATG8CL interaction with wild-type PexRD54 (35). The AIM motif disrupting PexRD54^Δ218AEIA^ and PexRD54^Δ298AEIA^ variants showed no binding to ATG8CL but retained a similar fold to PexRD54^Δ218^ and PexRD54^Δ298^ as judged by CD spectroscopy (Fig. 9).

We also tested whether the PexRD54^Δ218^ and PexRD54^Δ298^ deletions retained the ability to bind ATG8CL *in planta* by co-immunoprecipitation (co-IP) from *Nicotiana benthamiana* leaves transiently expressing these proteins following delivery of the genes by infiltration with *Agrobacterium tumefaciens* (agroinfiltration). In these assays both RFP-PexRD54 deletion mutants still interacted with ATG8CL (Fig. 8*C*). Full-length RFP-PexRD54 and the AIM motif disrupting variant RFP-PexRD54^378-AEIA-381^ were used as controls.

## Discussion

Understanding the mechanistic basis of translocated effector protein function in support of pathogen infection and colonization is a major focus of research in plant-microbe interactions. Such studies reveal how manipulation of host cell processes by pathogen-derived molecules can promote virulence and also identify plant systems, such as autophagy, whose importance in disease or general host cell physiology may be underappreciated. In a few cases, the structural basis for bacterial plant pathogen effector interaction with a host protein or peptide has been described (37–40). However, such studies of filamentous plant pathogen effectors are lacking. The *P. infestans* R*X*LR-type effector PexRD54 (PITG_09316) perturbs host-selective autophagy for the benefit of the pathogen via interaction with ATG8CL (35). Here, we focused on the biochemical and structural basis of PexRD54's interaction with ATG8CL to understand how the pathogen co-opts autophagic pathways.

Structural conservation in R*X*LR-type effectors from the oomycetes, in the absence of confidently assignable sequence similarity, has previously been established (24, 25). Although each of the five structurally conserved three-helical bundle (WY domain) repeats in PexRD54 adopts the same overall fold, they pack together to form a unique structure different from that of the two WY domain repeat effector ATR1 from *Hyaloperonospora arabidopsidis* (41). Detailed analysis of the PexRD54 structure suggests trajectories for the evolution of WY domain proteins through gain or loss of functional units presented on the N or C terminus of the core three-helical bundle. First, the minimal three helix WY domain fold seen in PexRD54 is found in *P. infestans* effector PexRD2 (24), but in other R*X*LR-type effectors of known structure an N-terminal helix is present resulting in a four-helical bundle. Interestingly, in PexRD54, the C-terminal helices of WY-1, WY-3, and WY-4 are positioned such that they also serve as N-terminal helical extensions to WY-2, WY-4, and WY-5 to build four-helical bundles as observed in AVR3a4 (42), AVR3a11, and ATR1. Second, in ATR1 the tandem repeats of the four helix bundle are separated by a fifth “linker” helix. When the first WY domain of ATR1 is overlaid on WY-5 of PexRD54, the fifth linker helix is positioned on the final helix of PexRD54 (*brown* in Fig. 3*A*). In both protein structures, this helix then serves to present the proximal regions, either a second WY domain as seen in ATR1 or the AIM region as seen in PexRD54. Finally, PexRD54:WY-3 does not have an N-terminal helix and does not form a four helical bundle. This correlates with a significant kink in the PexRD54 structure between WY-2 and WY-3. Each of these observations serves to highlight the plasticity of the WY-fold and how it can be utilized to deliver new template structures with the potential for functional diversification. It is interesting to note that conserved structure in the absence of confidently assignable sequence similarity is emerging as a recurring theme for filamentous plant pathogen effectors (43, 44).

Little is known about how plant autophagic pathways are controlled and manipulated by pathogens. The structure of ATG8CL bound to the PexRD54 AIM peptide revealed the fundamental mechanisms of AIM recognition by plant ATG8s are similar to those seen in other organisms. The two critical hydrophobic residues of the Ω*XX*Ψ motif, Trp and Val in PexRD54, are bound in two hydrophobic pockets on the surface of ATG8CL (Fig. 4*A*). Furthermore, our mutagenesis and peptide-binding studies confirm the important roles for these residues in the interaction. The identity of the residues to the N terminus of the AIM, which in other systems comprise acidic residues (11), do not seem to be important in this case. Previously, it was shown that the binding of PexRD54 to another ATG8 family member, ATG8IL, was weaker *in planta* and *in vitro*. These two proteins share 50% sequence identity. Interestingly, three amino acids are changed between ATG8CL and ATG8IL at the ATG8CL/PexRD54 AIM peptide interface: I33V, L56M, and Vl64I. ATG8CL Ile-33 is located at the base of the pocket that binds PexRD54 Trp-378, whereas ATG8CL Leu-56 and ATG8CL Val-64 are both located in the second hydrophobic pocket that faces PexRD54 Val-381. The interactions between ATG8s and AIM peptides are dominated by hydrophobic interactions, and the subtle changes delivered by these mutations may be responsible for the weaker binding affinity of ATG8IL over ATG8CL, although this remains to be tested *in vitro* and will be the subject of future work.

The previous study (35) and the work described here reveal the importance of the interaction between PexRD54 and ATG8CL, as mediated by the effector's C-terminal AIM region. This region includes only ∼3% of the amino acids downstream of the R*X*LR-dEER motif, but deletion of WY domains 1–4 does not significantly affect ATG8CL binding *in vitro* or *in planta*. This raises the following question. How do the five WY domains contribute to PexRD54 function? This effector has been shown to stimulate host autophagosome formation, and it was hypothesized that the pathogen exploits this for its own benefit in either promoting nutrient recycling or counteracting defense. Future work will address how the PexRD54 WY domains may contribute to autophagosome formation and/or act as a receptor to localize specific cellular cargo to autophagic pathways.

## Experimental Procedures

### Gene Cloning

All constructs were verified by DNA sequencing.

#### 

##### PexRD54

For protein expression in *E. coli*, DNA encoding PexRD54 residues Val-92 to Val-381 was amplified from RFP-PexRD54 (35) and cloned into pOPINA or pOPINS3C (45) by In-Fusion cloning (Clontech). The resultant vectors expressed PexRD54 protein without a fusion tag (pOPINA) or with the N-terminal His_6_-SUMO tag (pOPINS3C), respectively. DNA encoding PexRD54 residues Arg-219 to Val-381 was amplified from pOPINA-PexRD54 and cloned into pOPINS3C. DNA encoding PexRD54 residues Ser-299 to Val-381 was amplified from pOPINA-PexRD54 (and cloned into pOPINS3C) or from pOPINS3C-PexRD54 (and cloned into pOPINA). Single point mutants within the AIM region of PexRD54 were encoded within primers that were then used to amplify the full-length construct from pOPINS3C-PexRD54 followed by ligation into pOPINS3C. For protein expression *in planta*, DNA encoding PexRD54 residues Arg-219 to Val-381 or Ser-299 to Val-381 were amplified from RFP-PexRD54 and cloned into pENTR (ThermoFisher, UK). The expression constructs RFP-PexRD54^Δ218^ and RFP-PexRD54^Δ298^ were generated by Gateway LR reaction (Invitrogen) using the destination vector pH7WGR2 (N-terminal RFP fusion).

##### ATG8CL

For protein expression in *E. coli*, DNA encoding Met-1 to Phe-119 of ATG8CL was amplified from pOPINF-ATG8CL (35) and cloned into pOPINE (45), producing ATG8CL with a non-cleavable C-terminal His_6_ tag. DNA encoding Ser-5 to Asn-114 of ATG8CL was amplified from pOPINF-ATG8CL and cloned into pOPINF, expressing ATG8CL with a cleavable N-terminal His_6_ tag (called ATG8CL* hereafter). For probing the peptide array, DNA encoding ATG8CL residues Met-1 to Phe-119 was amplified from pOPINE-ATG8CL and cloned into pOG3182 (Oxford Genetics). DNA encoding the ATG8CL-GST fusion was amplified from ATG8CL-pOG3182 and cloned into pOPINE. The resultant pOPINE-ATG8CL-GST vector expressed ATG8CL protein with a non-cleavable C-terminal GST-His_6_ tag. For protein expression *in planta*, GFP-EV and GFP-ATG8CL constructs were described previously (35).

### Heterologous Protein Production and Purification

Purified proteins were concentrated and stored in 20 mm HEPES buffer, pH 7.5, containing 150 mm NaCl, except where stated.

#### 

##### PexRD54 and Its Variants

For analytical gel filtration and ITC, all PexRD54 proteins were produced using *E. coli* BL21-arabinose-inducible cells and purified as described previously (35). For SPR, the same purification protocol was followed, with the exception of the final gel filtration step, which used 20 mm HEPES, pH 7.5, 500 mm NaCl.

##### ATG8CL

ATG8CL, expressed from pOPINF, was produced in *E. coli* BL21(DE3) and purified as described previously (35). When produced from pOPINE, a single Ni^2+^-NTA capture step followed by gel filtration produced soluble protein. The same strategy was used for purifying pOPINE-ATG8CL-GST-His. For SPR, ATG8CL was purified using 20 mm HEPES, pH 7.5, 500 mm NaCl in the gel filtration step. For crystallization, pOPINF-ATG8CL* was expressed and purified as for pOPINF-ATG8CL, except auto-induction media were used to culture the *E. coli*.

##### PexRD54-ATG8CL Complex

For crystallization and SAXS analysis of the complex, pOPINA-PexRD54 and pOPINE-ATG8CL were co-transformed and expressed in BL21(DE3). Purification used the same protocol as for ATG8CL produced from pOPINE.

### Protein-Protein Interaction Studies

#### 

##### Analytical Gel Filtration

Analytical gel filtration chromatography was performed at 4 °C using a Superdex 75 10/300 column (GE Healthcare) pre-equilibrated in 20 mm HEPES, pH 7.5, 150 mm NaCl. 100 μl of sample was injected at a flow rate of 0.8 ml/min, and 0.5-ml fractions were collected for analysis. To study complex formation, proteins were mixed and incubated on ice for at least 1 h prior to loading.

##### Surface Plasmon Resonance

SPR experiments were performed at 18 °C using a BIAcore T200 system (GE Healthcare) and an NTA sensor chip (GE Healthcare). Protein samples were prepared in 20 mm HEPES, pH 7.5, 500 mm NaCl, and all the measurements were recorded in the same buffer at a flow rate of 30 μl/min. A single cycle kinetics approach was used to study the interaction between PexRD54 and ATG8CL. The NTA chip was activated by injecting 10 μl of 0.5 mm NiCl_2_ over flow cell 2, which was also used to immobilize His-tagged ATG8CL to a response level of 85 ± 2. Increasing concentrations of PexRD54 (20, 200, 600, 1000, and 2000 nm) were injected over flow cell 1 and 2 for 90 s. After the final injection, the dissociation was recorded for 300 s. Two startup cycles were run where the chip was activated and ATG8CL immobilized in the same manner, but buffer only was injected instead of PexRD54. This was subtracted to account for any dissociation of ATG8CL from the sensor chip. The sensor chip was regenerated by injecting 10 μl of 350 mm EDTA. The data were analyzed using BIAcore T200 BIAevaluation software (GE Healthcare) and then plotted with Microsoft Excel.

##### Isothermal Titration Calorimetry

Calorimetry experiments were recorded at 15 °C in 20 mm HEPES, pH 7.5, 150 mm NaCl, using an iTC200 instrument (MicroCal Inc.). The calorimetric cell was filled with 80 μm PexRD54 truncation (PexRD54^Δ218^ or PexRD54^Δ298^) and titrated with 0.8 mm ATG8CL from the syringe. A single injection of 0.5 μl of ATG8CL was followed by 19 injections of 2 μl each. Injections were made at 120-s intervals with a stirring speed of 750 rpm. The raw titration data were integrated and fitted to a one-site binding model using the MicroCal Origin software.

##### In Planta Co-immunoprecipitation

3–4-week-old *N. benthamiana* plants were used for transient expression experiments. T-DNA expression vectors encoding PexRD54 constructs, ATG8CL constructs, or empty vector were transformed into the *A. tumefaciens* GV3101 strain. Transformed agrobacteria were diluted in 5 mm MES, 10 mm MgCl_2_, pH 5.6, and mixed in 1:1 ratio to a final *A*_600_ of 0.2 prior to leaf infiltration.

*N. benthamiana* leaves transiently expressing proteins were harvested 2 days post-infiltration. Protein extraction, immunoprecipitation, and Western blotting analyses were performed as described previously (35). For blots shown in Fig. 8, mouse monoclonal single step GFP-HRP antibody (Santa Cruz Biotechnology) was used for GFP immunoblot experiments. For RFP blots, polyclonal RFP antibody (Invitrogen) was used as primary antibody and anti-rat HRP antibody (Sigma, UK) was used as secondary antibody.

### Crystallization, Data Collection, and Structure Solution

#### 

##### PexRD54 (in the Presence of ATG8CL)

For crystallization, the PexRD54-ATG8CL complex produced by co-expression was concentrated to 10 mg/ml in 20 mm HEPES, 150 mm NaCl, pH 7.5. Crystallization experiments used 4-μl hanging drops with a 2:1 protein/precipitant ratio. For data collection, crystals were grown in 18% PEG 10K, 0.1 m sodium acetate, pH 5.0, 0.18 m tri-ammonium citrate and transferred to a cryoprotectant solution consisting of 22% PEG 10K, 0.1 m sodium acetate, pH 5.0, 0.18 m tri-ammonium citrate and 10% ethylene glycol. To enable structure solution, crystals were soaked for ∼45 s in well solution supplemented with 500 mm potassium iodide and then cryoprotected as above.

Native and single wavelength anomalous diffraction x-ray data sets were collected at the Diamond Light Source, United Kingdom, beamline I02. The datasets were processed using the Xia2 pipeline (46), see Table 1. The structure was solved using the single wavelength anomalous diffraction approach with the data collected from the crystal soaked in potassium iodide solution. Iodide sites were identified with Phenix (47). These positions were used to estimate initial phases using PHASER EP from the CCP4 suite (48), followed by density improvement with PARROT (49). An initial model was built using BUCCANEER (50) followed by manual rebuilding and refinement using COOT (51) and REFMAC5 (52). Next, molecular replacement with Phaser, followed by the Phenix AutoBuild wizard, was used to produce an initial model of PexRD54 using the native x-ray data. The final model was produced through iterative rounds of refinement using REFMAC5 and manual rebuilding with COOT. Structure validation used the tools provided in COOT and MOLPROBITY (53).

##### ATG8CL

ATG8CL* mixed with a 3-fold molar excess of pentapeptide (Asp-Trp-Glu-Ile-Val) was incubated at 4 °C for 24 h and concentrated to 80 mg/ml in 20 mm HEPES, 150 mm NaCl, pH 7.5. Crystallization experiments used 2-μl sitting drops with a 1:1 protein/precipitant ratio. Crystals were produced in 0.2 m ammonium sulfate, 0.1 m Tris buffer, pH 8.0, and 36% PEG3350 and transferred to the precipitant solution with the addition of 10% ethylene glycol as a cryoprotectant. X-ray diffraction data were collected at the Diamond Light Source, UK, beamline I04, and the data were processed as above (Table 1). The structure was solved by molecular replacement using PHASER, as implemented in Phenix. The molecular replacement search model was generated by submitting the complete sequence of ATG8CL to the Phyre web server (54). Based on the solution, an initial model was produced using the AutoBuild wizard in Phenix. At this stage, clear electron density was apparent for the Asp-Trp-Glu-Ile-Val pentapeptide in both molecules of ATG8CL*. The final model was completed and validated as described for PexRD54. Data collection and refinement statistics for PexRD54 and ATG8CL are given in Table 1.

### SAXS Measurements, Data Processing, and Analysis

SAXS data were collected at the ESRF beamline BM29 (Grenoble, France (55, 56)) and at the Diamond Light Source, UK, beamline B21. For BM29, measurements were made at an energy of 12.5 keV, camera length of 2.81 m, and q range 0.003–5 nm^−1^. For B21, measurements were made at an energy of 12.4 keV, camera length of 4.018 m, and *q* range 0.004–3.8 nm^−1^. Measurements of 40 μl of protein solution at three different concentrations (0.5, 1.0, and 2.0 mg/ml European Synchrotron Radiation Facility (ESRF); 2.5, 5.0, and 10.0 mg/ml Diamond Light Source) were made for each sample (and buffer). Matched buffer measurements taken before and after every sample were averaged and used for background subtraction. Merging of separate concentrations and further analysis steps were performed manually using the ATSAS package (57, 58). DATCMP was used to exclude any individual frames showing signs of radiation damage using standard thresholds for the beamlines. For uncomplexed PexRD54, data collected at the ESRF were used for further analysis. Inspection of the SAXS data for the PexRD54-ATG8CL complex suggested the optimum dataset incorporated both the ESRF (low angles and wide angles) and DLS (mid-range angles) data, and these were merged manually. The forward scattering I(0) and radius of gyration (*R_g_*) for each particle were calculated from the Guinier approximation. The molecular mass of the samples was estimated using the Porod invariant (59) and the maximum particle sizes (*D*_max_) were determined from the pair distribution function computed by GNOM (60) using PRIMUS (61). For both PexRD54 and the PexRD54-ATG8CL complex, 40 *ab initio* models were calculated using DAMMIN (62). DAMSEL compared these models and calculated a mean normalized spatial discrepancy (NSD) of 0.545 ± 0.02 for PexRD54 (discarding only one model with NSD > mean ± 2× S.D.), and a mean NSD of 0.635 ± 0.03 for PexRD54-ATG8CL complex (no models discarded). DAMSEL also identified the most probable (lowest NSD) model. All non-discarded models were aligned, averaged, and compared using DAMSUP, DAMAVER, and DAMFILT in ATSAS for analysis. Rigid body modeling of the PexRD54-ATG8CL complex was achieved with CORAL (36), with the inclusion of the missing residues and linker region that were not visible in the electron density maps of PexRD54 or ATG8CL. The fits of the most probable *ab initio* models to the experimental data were calculated by DAMMIN, the theoretical scattering of PexRD54 was calculated with CRYSOL (63), and the fit of the PexRD54-ATG8CL complex was as calculated by CORAL. Rigid body models of PexRD54 and the PexRD54-ATG8CL complex were overlaid with the *ab initio* models using SUPCOMB (64) and viewed in PyMOL.

### Peptide Library

The PexRD54-AIM peptide library was synthesized by Kinexus (Vancouver, Canada) and included 200 peptides where each amino acid in the last 10 amino acids of PexRD54 was changed to every other amino acid. The peptides were spotted on cellulose membrane (Invatis, Germany) with free C termini. Peptide interactions with the ATG8CL-GST-His fusion protein were determined as described previously. The membrane was blocked with 5% (w/v) nonfat dried milk in TBS-T, washed with TBS-T, and overlaid with 1 μg/ml purified ATG8CL-GST-His fusion protein for 2 h at room temperature. The membrane was washed in TBS-T, and bound proteins were detected with HRP-conjugated anti-GST antibody (1:5000) (RPN1236; GE Healthcare, UK).

### Circular Dichroism Spectroscopy

CD spectroscopy experiments were performed using a Chirascan-Plus CD spectrophotometer (Applied Photophysics). Purified proteins in 20 mm HEPES, pH 7.5, 150 mm NaCl at a concentration of at least 10 mg/ml were diluted to 0.2 mg/ml in 20 mm di-potassium phosphate, pH 7.2. CD measurements were carried out in a quartz glass cell with a 0.5-mm path length. To obtain overall CD spectra, wavelength scans between 190 and 260 nm were collected at 15 °C using a 2.0-nm bandwidth, 0.5-nm step size, and time per point of 1 s. The data were collected over four accumulations and averaged. The raw data in millidegree units were corrected for background and converted to mean residue molar ellipticity.

## Author Contributions

A. M., R. K. H., Y. F. D., T. O. B., S. K., and M. J. B. designed the research; A. M., R. K. H., Y. F. D., N. T., and E. Z. performed the experiments; K. B. provided reagents and analytic tools; A. M., R. K. H., Y. F. D., A. R., T. O. B., and M. J. B. analyzed the data; A. M., R. K. H., and M. J. B. wrote the paper with editorial input from all authors.

## Supplementary Material

Supplemental Data

## Footnotes

3 The abbreviations used are:

AIM: ATG8-family interacting motif
SPR: surface plasmon resonance
NTA: nitrilotriacetic acid
RFP: red fluorescent protein
ITC: isothermal titration calorimetry
SAXS: small angle x-ray scattering
NSD: normalized spatial discrepancy.
