# Supplementary material for: Structural Basis of Host Autophagy-related Protein 8 (ATG8) Binding by the Irish Potato Famine Pathogen Effector Protein PexRD54
Source: J Biol Chem. 2016 Jul 25;291(38):20270–82. doi: 10.1074/jbc.M116.744995 (PMC5025708; doi:10.1074/jbc.M116.744995)
Supplement: Supplemental Data [file supp_291_38_20270__index.html]

Structural basis of host Autophagy-related protein 8 (ATG8) binding by the Irish potato famine pathogen effector protein PexRD54 — Structural Basis of Host Autophagy-related Protein 8 (ATG8) Binding by the Irish Potato Famine Pathogen Effector Protein PexRD54 — Structure/Function of PexRD54 — Supplemental Data 

# Structural Basis of Host Autophagy-related Protein 8 (ATG8) Binding by the Irish Potato Famine Pathogen Effector Protein PexRD54

## Supplemental Data

- Supplementary Video 1 (.mpg, 27.3 MB) - Video showing the structure of PexRD54
